# Bone Loss Triggered by the Cytokine Network in Inflammatory Autoimmune Diseases

**DOI:** 10.1155/2015/832127

**Published:** 2015-05-04

**Authors:** Dulshara Sachini Amarasekara, Jiyeon Yu, Jaerang Rho

**Affiliations:** Department of Bioscience and Biotechnology, College of Biological Sciences and Biotechnology, Chungnam National University, 99 Daehak-ro, Yuseong-gu, Daejeon 305-764, Republic of Korea

## Abstract

Bone remodeling is a lifelong process in vertebrates that relies on the correct balance between bone resorption by osteoclasts and bone formation by osteoblasts. Bone loss and fracture risk are implicated in inflammatory autoimmune diseases such as rheumatoid arthritis, ankylosing spondylitis, inflammatory bowel disease, and systemic lupus erythematosus. The network of inflammatory cytokines produced during chronic inflammation induces an uncoupling of bone formation and resorption, resulting in significant bone loss in patients with inflammatory autoimmune diseases. Here, we review and discuss the involvement of the inflammatory cytokine network in the pathophysiological aspects and the therapeutic advances in inflammatory autoimmune diseases.

## 1. Introduction

Bone is the main calcified tissue of vertebrates and serves multiple functions including mechanical support, protection, and storage [1]. The composition of bone is approximately 10% cells, 60% mineral crystals (crystalline hydroxyapatite), and 30% organic matrix [2]. Bone is continuously maintained by the process of bone remodeling through clusters of bone-resorbing osteoclasts and bone-forming osteoblasts [1, 3]. During bone remodeling, old or damaged bone is removed by osteoclasts and replaced by new bone formed by osteoblasts over several weeks [1, 3].

Osteoblasts are of mesenchymal origin and function primarily as bone-forming cells [1, 4]. Osteoblasts secrete the organic matrix, which predominantly contains collagen, and induce calcification during the process of new bone formation [5]. During bone remodeling, osteoblasts rebuild the bone matrix in regions where the bone has been resorbed by osteoclasts [1, 4]. The differentiation and function of osteoblasts are regulated by the activation of transcription factors (i.e., Runx-2/Cbfa-1, osterix (Osx), TAZ, and Atf4) [6–9], growth factors (i.e., tumor growth factor-*β* (TGF-*β*), bone morphogenetic proteins (BMPs), Wnt, and vascular endothelial growth factor) [10–13], cytokines (i.e., interleukin-1 (IL-1), IL-6, and tumor necrosis factor-*α* (TNF-*α*)), and interactions with various matrix proteins (i.e., collagen type I, biglycan, laminin, and fibronectin) [14, 15]. At the end of the bone-forming phase during bone remodeling, osteoblasts incorporate into the bone as osteocytes and the rest either remain on the bone surface as lining cells or undergo apoptosis [5, 16].

Osteocytes are former osteoblasts that become trapped during the process of bone deposition and remain regularly distributed throughout the mineralized bone matrix. These cells comprise more than 90% of bone cells within the matrix or on bone surfaces [17]. Osteocytes are the primary mechanosensory cells that act as regulators of mineral metabolism during bone remodeling [17]. Studies have revealed that osteocytes can send signals of bone resorption to osteoclasts during bone remodeling [17, 18]. Osteoclasts, the sole bone-resorbing cells, are multinucleated giant cells that are derived from mononuclear cells of the monocyte/macrophage lineage following stimulation by two essential factors: the macrophage colony-stimulating factor (M-CSF) and the receptor activator of nuclear factor-kappa B (RANK) ligand (RANKL) [1, 3, 4].

**Figure 1:**
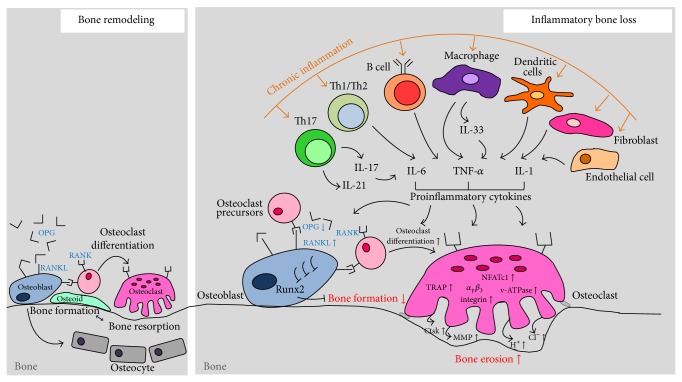
The role of inflammatory cytokine network in inflammatory bone loss. Bone remodeling is tightly regulated by the balanced action between bone-forming osteoblasts and bone-resorbing osteoclasts. In chronic inflammatory condition, inflammatory cytokine networks induce an uncoupling of bone formation and resorption that result in significant inflammatory bone loss. RANK: receptor activator of nuclear factor *κ*B. RANKL: RANK ligand. OPG: osteoprotegerin. Runx2: runt-related transcription factor 2. TRAP: tartrate-resistant acid phosphatase. NFATc1: nuclear factor of activated T cells cytoplasmic 1. v-ATPase: vacuolar-type H^+^-ATPase. MMP: matrix metalloproteinase. Ctsk: cathepsin K.

The process of bone remodeling depends on the tight coupling of bone formation and bone resorption to ensure that there is no net change in the bone mass and to maintain the quality after each remodeling cycle [1, 3, 4]. An imbalance in this process is closely linked to various types of bone diseases, such as osteoporosis, osteopetrosis, periodontitis, and rheumatoid arthritis (RA) [19]. Osteoporosis is a skeletal disorder characterized by compromised bone strength, predisposing patients to an increased risk of fracture [20]. Osteoporosis was first considered to be an age-related disorder characterized by low bone mass and increased bone fragility, thereby putting the patient at risk of fractures. However, over time, it has come to be viewed as a heterogeneous condition that can occur at any age and its etiology is attributed to various endocrine, metabolic, and mechanical factors [19]. Studies have reported an increased risk of developing osteoporosis in patients with various inflammatory conditions [1–4]. Inflammation is characterized by the activation of several cell populations of the innate and adaptive immune system that produce inflammatory cytokines [21]. Inflammation perturbs normal bone homeostasis and is known to induce bone loss because it promotes both local cartilage degradation and local and systemic bone destruction by osteoclasts and inhibits bone formation by osteoblasts (Figure 1).

Inflammatory joint diseases share in common the presence of an inflammatory process that targets the joints, with adverse effects on structure and function [22]. RA is one of the most common autoimmune* diseases* that results in chronic* inflammation* of the* joints* [23]. Autoimmune diseases are characterized by impaired function and destruction of tissues caused by the presence of autoantibodies due to abnormally activated lymphocytes and nonlymphoid cells, such as macrophages, dendritic cells, and fibroblasts [24, 25]. Dysregulation of inflammatory or anti-inflammatory cytokine production or action is reported to play a central role in the pathogenesis of autoimmune diseases such as RA, ankylosing spondylitis (AS), inflammatory bowel disease (IBD), and systemic lupus erythematosus (SLE) [26–32]. Studies have revealed that therapeutic approaches using inflammatory/anti-inflammatory cytokines, including neutralizing antibodies (i.e., anti-TNF-*α*, anti-IL-6, and anti-IL-17), soluble receptors/inhibitors (i.e., TNF receptor, IL-1 receptor, IL-17 receptor, and IL-6 receptor inhibitor), and anti-inflammatory cytokines (i.e., IL-10 and IL-27), have been successful in controlling the progression of autoimmune diseases [33–37]. These studies have demonstrated a possible link between chronic inflammation and the pathogenesis of autoimmune diseases. Moreover, chronic inflammatory autoimmune diseases are frequently associated with bone destruction [38]. Bone loss is commonly observed in inflammatory joint diseases such as RA and AS [22]. Studies have also found an increase in bone loss and fractures with low BMD in individuals with SLE and IBD [38].

Although a large number of studies have focused on inflammatory autoimmune diseases over the past 10 years, the role of the inflammatory cytokine network involved in bone loss in patients with inflammatory autoimmune diseases has not been well addressed. Therefore, in this review, we will provide an overview of the interaction between inflammatory autoimmune diseases and bone destruction through the regulation of the inflammatory cytokine network.

## 2. Methodology

We performed an extensive internet search for scientific articles indexed in the PubMed/Medline database over the past 15 years using the following keywords: bone loss, osteoporosis, autoimmunity, rheumatoid arthritis, ankylosing spondylitis, inflammatory bowel disease, and systemic lupus erythematosus. We specifically focused on how bone loss and fracture risk are implicated in inflammatory autoimmune diseases.

## 3. Rheumatoid Arthritis (RA)

RA is a chronic autoimmune inflammatory disease characterized by the production of two main autoantibodies, rheumatoid factor and anticitrullinated peptide antibody, against common autoantigens that are widely expressed outside the joints, thereby resulting in local bone erosion, joint space narrowing, and extra-articular manifestations [23, 39]. In severe cases, RA can lead to periarticular osteopenia, systemic osteoporosis, and systemic bone erosion [40]. Disturbance of bone homeostasis in RA patients is driven by the cellular action of osteoclasts [41]. The enhanced osteoclast formation and activation is due to the increased accumulation of osteoclastogenic factors in the inflamed synovium [42–45]. In RA, elevated inflammatory cytokines have been implicated in bone destruction through recruitment of osteoclast precursors to the bone environment, where they differentiate into mature osteoclasts [46–48]. These inflammatory cytokines, such as TNF-*α*, IL-1, IL-6, IL-7, and IL-17, are responsible for the overexpression of RANKL and decreased levels of osteoprotegerin (OPG), a decoy receptor of RANK. This perturbation leads to an imbalance in the RANKL/OPG ratio, thereby increasing osteoclast differentiation (also known as osteoclastogenesis) [42, 49–52]. However, levels of anti-inflammatory cytokines such as IL-10, IL-13, and TGF-*β* have been reported to be present in significant amounts in RA joints [53, 54]. These anti-inflammatory cytokines have a negative effect on the joint destruction and inflammation associated with RA [55].

The role of TNF-*α* in arthritic bone destruction has been demonstrated in several experimental models and confirmed by clinical trials [56]. TNF-*α* enhances osteoclastogenesis through elevated expression of RANKL in the osteoblast [57]. Moreover, TNF-*α* induces the expression of the osteoclast-associated receptor (OSCAR), a key costimulatory molecule in osteoclastogenesis, on monocytes in RA patients [58]. TNF-*α* is also involved in osteoclastogenesis through modulation of the Wnt signaling pathway, although Wnt signaling is considered to be a key regulatory pathway for bone formation by osteoblasts [59]. In RA, TNF-*α* is a strong inducer of the Wnt antagonist Dickkopf-1 (Dkk-1) expression [60]. Dkk-1 impairs local bone formation through the inhibition of Wnt signaling by binding to low density lipoprotein-coupled receptor related protein-5/6 [61]. The blockade of Dkk-1 inhibits local bone resorption by reducing osteoclast numbers through the downregulation of OPG expression in the joints; this is further compounded because OPG regulates Dkk-1 expression through a feedback loop [60]. Consequently, the enhanced levels of Dkk-1 induced by TNF-*α* promote bone resorption by increasing the RANKL/OPG ratio but also block bone formation and repair in the diseased joint [62]. Furthermore, TNF-*α* is reported to directly inhibit osteoblast differentiation and bone nodule formation [63]. The transcription factors Runx-2/Cbfa-1 and Osx, which are critical regulators of osteoblast differentiation, are reported to be inhibited by TNF-*α*, thereby decreasing osteoblast differentiation and inhibiting bone formation [13]. Because TNF-*α* is the most important cytokine involved in both pathogenesis and joint inflammation associated with RA, TNF-*α* blockers were the first class of biologics used in RA [41]. A study by Smolen et al. showed that TNF-*α* blockers had a beneficial effect on inflammatory disease activity and joint degradation, achieving high rates of sustained clinical remission by preventing radiographic damage in RA [64]. Moreover, studies have reported that TNF-*α* antibodies can decrease systemic bone loss and increase bone mineral density indicating that anti-TNF-*α* can be used against systemic osteoporosis and osteopenia [65, 66].

IL-1 is a key regulatory cytokine in mouse models of inflammatory arthritis. Overexpression of IL-1*α* or IL-1*β* or deletion of the IL-1 receptor antagonist (IL-1Ra) leads to the development of arthritis with cartilage and bone destruction [48, 67]. IL-1 upregulates the production of RANKL, resulting in an imbalance in the synovial RANKL/OPG ratio [51, 68, 69]. In TNF-transgenic mice lacking IL-1 signaling, cartilage destruction is completely blocked and bone destruction partly reduced despite the presence of synovial inflammation, indicating that TNF-induced local bone destruction and systemic inflammatory bone loss are largely dependent on IL-1 [48]. Moreover, it is evident that TNF-induced synthesis of RANKL is inhibited by IL-1Ra [51]. In addition to IL-1 and TNF, IL-6 is another key proinflammatory cytokine involved in the pathogenesis of RA [70]. IL-6 stimulates the synthesis of RANKL by osteoblasts and promotes the development of T helper 17 (Th17) cells together with TGF-*β* and IL-1 [71]. Studies have shown that the IL-6 antagonist tocilizumab has a beneficial effect on joint destruction and disease progression in RA patients [72, 73]. In mouse RA models, inflammatory cytokines such as IL-1*β*, TNF-*α*, and IL-6 activate the signal transducer and activator of transcription 3 (STAT3) either directly or indirectly in murine osteoblasts and fibroblasts [68]. Studies have shown that STAT3 is the key mediator of both chronic inflammation and joint destruction in RA [68]. STAT3 activation induces the expression of RANKL [68, 74]. Therefore, STAT3 inhibition is also considered to be effective in treating RA.

IL-17 is the most recently described subclass of inflammatory cytokines. IL-17 induces the secretion of proinflammatory cytokines (i.e., TNF-*α*, IL-1*β*, and IL-6) and chemokines (i.e., CXCL1/KC/GRO*α*, CXCL2/MIP2*α*/GRO*β*, CXCL8/IL-8, CCL2/MCP1, and CCL20/MIP-3*α*) from cartilage, synoviocytes, macrophages, and bone cells [75–81]. These elevated inflammatory cytokines and chemokines serve to activate and recruit neutrophils, macrophages, and lymphocytes to the inflamed synovium, thereby enhancing synovial inflammation [82]. Intra-articular injection of recombinant IL-17 also results in joint inflammation and damage [79, 83]. Interestingly, IL-17 activity is synergistically increased when combined with proinflammatory cytokines such as TNF-*α*, IL-1*β*, and IL-6 [84, 85]. Moreover, IL-17 contributes to extensive cartilage and bone erosion in the advanced stages of RA by inducing the expression of RANKL, matrix metalloproteinases (MMPs), prostaglandin E2, and cyclooxygenase-2 [83, 86, 87]. The role of IL-17 as a potent stimulator of osteoclastogenesis in RA patients was first demonstrated by Kotake et al. [46]. IL-17 regulates osteoclastogenesis both directly and indirectly through osteoblasts/stromal cells, although the direct effect of IL-17 on osteoclast precursors is still controversial [87–89]. IL-17 induces RANKL expression from osteoblasts, synovial cells, and mesenchymal cells, and the increased RANKL/OPG ratio results in local or systemic bone destruction through enhancement of osteoclastogenesis [42, 46, 90]. Moreover, IL-17-producing Th17 cells, a subset of RANKL-expressing CD4^+^ T cells, are involved in bone destruction through the function of osteoclastogenic helper T cells [87, 91]. In animal model studies, therapeutic approaches using IL-17 antibodies or a soluble IL-17 receptor have resulted in significant suppression of joint inflammation and bone erosion through downregulation of synovial RANKL and inflammatory cytokine expression [92–94]. Therefore, blocking IL-17, the IL-17 receptor (IL-17R), or its inducers (i.e., IL-23 and IL-6) can be used as a putative treatment method for RA.

In conclusion, bone destruction in RA is caused by a complex network of inflammatory cytokines, resulting in the* chronic inflammation* of the synovium. These studies have revealed several promising targets for the treatment of inflammatory bone loss in RA. In this respect, the initiation of biological therapies targeting inflammatory cytokines and/or lymphocyte activation has modified RA therapy not only by blocking local and systemic inflammatory cascades but also by providing beneficial effects against bone and joint destruction.

## 4. Ankylosing Spondylitis (AS)

AS is a systemic rheumatic disease characterized by chronic inflammation that chiefly affects the sacroiliac joints and the spine, whereas RA primarily affects the synovial membrane [95, 96]. One of the main features of structural damage in AS is bony ankyloses characterized by excessive bone formation that leads to the formation of bone spurs, such as syndesmophytes and enthesophytes, that contribute to ankylosis of the joints and poor physical function [96]. Moreover, the excessive loss of trabecular bone in the center of the vertebral body causing osteopenia or osteoporosis and leading to vertebral fractures with increased spinal deformity has been documented in AS patients [97].

TNF-*α* is a pivotal cytokine fueling inflammation in AS [96, 98]. TNF-*α*-targeted therapies have influenced short-term control of the disease by limiting the symptoms caused by inflammation, which translates into better physical function and quality of life [96]. However, little or no effect on structural remodeling is achieved [99]. The elevated levels of IL-1 and IL-6 in the serum and in the sacroiliac joints of AS patients are also implicated in AS [32, 100]. However, antibody therapies blocking IL-6R signaling with tocilizumab or sarilumab failed to show clinical efficacy in a phase II clinical trial with AS patients, suggesting that IL-6 is not a pivotal inflammatory cytokine in the pathogenesis of AS [101, 102].

The involvement of Th17 cells in the promotion of the inflammatory process in AS patients is shown by the significantly elevated levels of Th17 cells in the peripheral blood of patients with AS [103, 104]. IL-17 and IL-23 are also high in the serum of AS patients [30]. Moreover, antibody therapies such as blocking IL-17 with secukinumab were shown to significantly downregulate the signs, symptoms, and objective parameters of inflammation in a phase II clinical trial in AS patients [105]. Currently, phase III clinical trials consisting of antibody therapy with secukinumab in AS patients are ongoing [106].

Previous studies have documented that the serum level of RANKL is higher in AS patients and that the expression of RANKL is increased on CD4 and CD8 T cells in AS patients [107]. Inflammatory cytokines including IL-1, IL-6, TNF-*α*, and IL-17 can stimulate the expression of the soluble form of RANKL, which imbalances the RANKL/OPG ratio in AS patients [38]. The increased RANKL/OPG ratio thus promotes osteoclast differentiation, resulting in the bone destruction that is characteristic of AS [108, 109].

## 5. Inflammatory Bowel Disease (IBD)

IBD primarily refers to Crohn's disease and ulcerative colitis [110]. Crohn's disease can affect any part of the gastrointestinal tract, and classically presents with fatigue, prolonged diarrhea with or without gross bleeding, abdominal pain, weight loss, and fever [111]. Ulcerative colitis is limited to the colon area; common symptoms include rectal bleeding, frequent stools, mucus discharge from the rectum, tenesmus, and lower abdominal pain [111]. Crohn's disease is reported to be associated with Th1 cytokines IL-2, IL-17, interferon-*γ* (IFN-*γ*), and TNF-*α*, while ulcerative colitis is associated with Th2 cytokines, such as IL-4, IL-5, and IL-13 [112]. Therefore, Th1, Th2, and Th17 cells seem to be broadly involved in the pathogenesis of IBD through the regulation of inflammatory cytokine network. Interestingly, low bone matrix density (BMD) (defined as osteopenia or osteoporosis) is a known* chronic* complication of IBD [113]. Although IBD is not the sole risk factor for developing osteoporotic bone loss, it appears to be related to other known osteoporosis risk factors such as age, sex, body mass index, and medication [113]. Thus, the acceleration of the development of new biological drugs for IBD requires expanded insights into understanding the physiology, mechanism, and pathogenesis of IBD.

The principal mechanisms behind reduced BMD in IBD patients are still not completely understood, but a complex network of inflammatory cytokines that influence bone destruction has been reported [110, 113]. Mucosal and systemic concentrations of many pro- and anti-inflammatory cytokines are elevated in IBD patients [114]. In particular, the enhanced production of proinflammatory cytokines such as TNF-*α*, IL-1*β*, and IL-6 is well documented in IBD patients [115, 116]. These proinflammatory cytokines stimulate bone resorption by osteoclasts through the induction of RANKL expression [1, 4]. Interestingly, anti-TNF-*α* therapy has been shown to improve markers of bone metabolism and BMD (i.e., osteocalcin, alkaline phosphatase, and P1NP) by decreasing serum OPG levels in IBD patients [117–120]. The increased RANKL/OPG ratio is known to promote osteoclast differentiation and bone destruction in IBD patients [121].

IL-17-producing Th17 cells are considered to be a new subset of cells that is critical for the reduced BMD in chronic IBD patients [122]. Th17 cells produce IL-17, IL-17F, IL-21, and IL-22; IL-17, IL-21, and IL-22 levels were reported to be markedly* elevated* in IBD patients [122]. IL-21 secreted by Th17 cells is one of the crucial cytokines involved in the pathogenesis of IBD via the induction of Th1 and Th17 immune responses in the gut [110]. Studies have shown that IL-21-deficient mice were resistant to Th1/Th17 cell-driven colitis [123, 124]. Correspondingly, IL-17 and IFN-*γ* production by activated lamina propria mononuclear cells from IBD patients were inhibited by an IL-21 blocking antibody [123, 124].

IL-33, a new member of the IL-1 family, is a ligand for the IL-1 receptor-related protein (ST2) that is anticipated to be essential for the induction of Th2 immune responses [125]. Enhanced IL-33 levels are closely associated with IBD, particularly in ulcerative colitis patients [126]. Correspondingly, the inhibition of IL-33 signaling through anti-ST2 antibody treatment attenuates the severity of arthritis in an animal RA model [127]. Furthermore, IL-33 stimulates human osteoclast differentiation through the activation of ST2 receptor signaling [128]. Thus, it may be possible that IL-33 directly or indirectly regulates RANKL- or Th2 response-mediated bone loss in IBD.

Therapeutic anti-TNF-*α* antibodies such as infliximab and adalimumab are used for the treatment of severe cases of IBD [129, 130]. However, approximately one-third of the patients benefit minimally or not at all from this treatment [129, 130]. This could indicate that, among patients with IBD, nonresponders to anti-TNF therapy are more likely to have an inflammatory response mediated by other proinflammatory cytokines, such as IL-1*β*, IL-6, IL-17, and IFN-*γ*. Therefore, new drugs targeting other inflammatory cytokines could potentially be useful for treating IBD patients who do not respond to anti-TNF therapy [131].

## 6. Systemic Lupus Erythematosus (SLE)

SLE is an autoimmune disease that predominantly affects young women and is characterized by immunological hyperactivity and multiorgan damage. The exact causative factors of SLE are still unknown [132]. Unrestricted hyperactivation of the immune system may lead to the overproduction of autoantibodies, immune complex deposition, and inflammatory cytokine release, eventually resulting in the SLE phenotype [132]. In particular, the dysregulation of T/B cell activation leads to the production of autoantibodies such as anti-double-stranded DNA, anti-Ro (SS-A), anti-La (SS-B), anti-Smith (Sm), and anti-ribonucleoprotein (RNP) in SLE patients [133]. Autoantibodies bound with antigens are deposited in organs, thereby causing chronic inflammation and tissue damage [132].

The abnormal expression of various inflammatory cytokines due to chronic inflammation induces an imbalance among different immune cell subsets, such as Th1/Th2 and Th17/regulatory T (Treg) cells; this imbalance plays a crucial pathogenic role in SLE [132]. TNF-*α* has been implicated in SLE murine models [26], and elevated serum TNF-*α* levels are observed in SLE patients, similar to the other inflammatory autoimmune diseases discussed here [134]. However, the therapeutic effects of TNF-*α* blockers in SLE patients are still controversial [135]. Abnormal IL-6 levels were also observed in both serum and local tissues in patients with SLE [136]. The dominant role of IL-6 in SLE pathogenesis is to accelerate autoantibody production by promoting the proliferation of autoreactive B cells [132]. The autoantibody production induced by IL-6 is indirectly mediated by IL-21 produced by CD4^+^ T cells [137]. Interestingly, it has been reported that IL-6 produced by dendritic cells inhibits Treg cell function in mouse SLE models [138]. Thus, IL-6 is implicated as the most important inflammatory cytokine in the pathogenesis of SLE, and antibody therapies blocking IL-6 receptor signaling with tocilizumab are reported to be effective in treating SLE [139].

IL-17 is a proinflammatory cytokine with multiple functions in the regulation of tissue inflammation [132]. An increased number of Th17 cells and elevated serum IL-17 levels are reported in SLE patients [140, 141]. In SLE patients, IL-17 seems to facilitate both T cell activation and infiltration into tissues via the expression of intercellular adhesion molecule-1 (ICAM-1) and B cell activation and antibody production in combination with B-cell-activating factor (BAFF) [140, 142]. A strong correlation between IL-17 and IL-23 levels in SLE patients suggests that IL-23 contributes to SLE severity by activating Th17 cells [143]. Moreover, the IL-23/IL-17 activation pathway is closely associated with increased immunoglobulin deposition and complement activation in the kidney in mouse SLE models [144]. IL-17 has not been therapeutically targeted in SLE patients to date, but data from recent clinical trials in other inflammatory autoimmune diseases such as and Crohn's disease can partially inform us about the efficacy and safety of blocking IL-17 either directly or indirectly by targeting IL-23 in SLE patients [145].

Since the first reported association between type I IFN and SLE in 1979 [146], many reports have implicated elevated levels of serum IFN-*α* in SLE [147]. Plasmacytoid dendritic cells (pDCs), which are abundant in the skin and lymph nodes, are reported to be the primary sources of IFN-*α* in SLE patients. The IFN signature produced by pDCs can promote the pathogenesis of SLE by enhancing autoantibody production and activating Th17 cells to secrete cytokines [148–150]. Considering the essential role of type I IFN in SLE, more than five monoclonal antibodies specific for different IFN-*α* isoforms or their receptors are in different clinical phases of testing [151].

There seems to be a high prevalence of osteoporosis in SLE patients, but the prevalence frequencies differ widely as a consequence of differences in body mass, age, sex, ethnicity, disease severity, and medication use [152]. Glucocorticoid use, longer disease duration due to chronic inflammation, neuropsychiatric disease complications, and previous fractures were identified as associated factors for SLE-related osteoporotic fractures [152]. Although the direct correlation between inflammatory cytokine levels and bone defects in SLE patients remains unclear, bone destruction in SLE patients is thought to be the result of accelerated osteoclastogenesis induced by proinflammatory cytokines [153]. The increased level of proinflammatory cytokines such as TNF-*α*, IL-1, IL-6, and IL-17 in SLE patients might result in an RANKL/OPG imbalance by enhancing RANKL induction, leading to accelerated osteoclastogenesis. Interestingly, increased levels of oxidized low density lipoproteins (LDL) have been reported in SLE patients [154]. The enhanced oxidized LDL can induce T cell activation, thereby sequentially inducing RANKL expression and TNF-*α* production [154–156]. Furthermore, a recent study by Tang et al. has shown that impaired osteoblast differentiation through the inhibition of the BMP/Smad pathway by activated NF-*κ*B signaling plays a role in the pathology of osteoporosis in SLE patients [153]. The number of Th17 cells and IL-17 levels are elevated in the serum of many SLE patients [140, 141]. Although the exact role of IL-17 in bone destruction in SLE patients remains unclear, IL-17 may affect bone remodeling through its effects on both osteoblasts and osteoclasts as discussed above; IL-17 can induce bone loss by mediating an imbalance in RANKL/OPG via the expression of RANKL in osteoblasts or activated T cells and can act in synergy with TNF-*α* or chemokines to influence osteoclast resorption [46, 75, 84, 85, 157].

## 7. Discussion 

Bone remodeling is a highly coordinated process that involves bone resorption and formation, which are essential for repairing damaged bones and maintaining mineral homeostasis. However, in chronic inflammatory conditions, the inflammatory cytokine network induces an uncoupling of bone formation and resorption that results in significant inflammatory bone loss. In particular, inflammatory cytokines such as IL-1, IL-6, IL-17, and TNF-*α* are involved in the pathogenesis of inflammatory autoimmune diseases of interest. However, the effects of inflammatory cytokines on inflammatory bone loss and in the pathogenesis of inflammatory autoimmune diseases are more complicated. As discussed in this review, bone loss in inflammatory autoimmune diseases may be caused by direct or indirect effects with complicated mechanisms by inflammatory cytokines or the inflammatory cytokine network in chronically inflamed tissues. Therefore, drugs targeting multiple cytokines could be an effective strategy for disease prevention and reducing disease progression. Because most inflammatory cytokines are involved in bone damage though inducing an imbalance in RANKL/OPG, focusing on OPG or RANKL management may be a better strategy than focusing on inhibiting a single cytokine.

Inflammatory autoimmune diseases continue to be a mounting public health concern worldwide. The cost and the social burden associated with these diseases, while being difficult to pin down accurately, are increasing. To stick with the saying “easing the burden: solutions for the future,” it is imperative to accelerate the development of new treatment options for these diseases. A better understanding of the mechanisms by which the inflammatory cytokine network elicits chronic inflammation in autoimmunity will provide new therapeutic approaches to reduce bone destruction in inflammatory autoimmune diseases.

## Abbreviations

RA: Rheumatoid arthritis
AS: Ankylosing spondylitis
IBD: Inflammatory bowel disease
SLE: Systemic lupus erythematosus
BMD: Bone mineral density
RANKL: Receptor activator of nuclear factor-kappa B ligand
M-CSF: Macrophage colony-stimulating factor
OPG: Osteoprotegerin
TNF-*α*: Tumor necrosis factor-*α*
IL: Interleukin
Dkk-1: Dickkopf-1
IL-1Ra: IL-1 receptor antagonist
STAT3: Signal transducer and activator of transcription 3
MMPs: Matrix metalloproteinases
IL-17R: IL-17 receptor
IFN-*γ*: Interferon-*γ*
Treg cells: Regulatory T cells
ICAM-1: Intercellular adhesion molecule-1
BAFF: B-cell-activating factor
pDCs: Plasmacytoid DCs
OSCAR: Osteoclast-associated receptors
Osx: Osterix
BMPs: Bone morphogenetic proteins.
